# Author Correction: The unusual quadruple bonding of nitrogen in ThN

**DOI:** 10.1038/s41467-023-44262-3

**Published:** 2023-12-20

**Authors:** Zejie Fei, Jia-Qi Wang, Rulin Tang, Yuzhu Lu, Changcai Han, Yongtian Wang, Jing Hong, Changwu Dong, Han-Shi Hu, Xiao-Gen Xiong, Chuangang Ning, Hongtao Liu, Jun Li

**Affiliations:** 1 Key Laboratory of Interfacial Physics and Technology, Shanghai Institute of Applied Physics, Chinese Academy of Sciences, Shanghai, 201800 China; 2 Department of Chemistry, Tsinghua University, Beijing, 100084 China; 3 College of Science, Beijing Forestry University, Beijing, 100083 China; 4 Department of Physics, State Key Laboratory of Low Dimensional Quantum Physics, Collaborative Innovation Center of Quantum Matter, Tsinghua University, Beijing, 100084 China; 5 University of Chinese Academy of Sciences, Beijing, 100049 China; 6 Sino-French Institute of Nuclear Engineering and Technology, Sun Yat-sen University, Zhuhai, 519082 China

**Keywords:** Decision, Reward, Motivation

Correction to: *Nature Communications* 10.1038/s41467-023-43208-z, published online 24 November 2023

In this article the affiliation details for Xiao-Gen Xiong were incorrectly given as ‘Sun Yat-sen University, Sino-French Institute of Nuclear Engineering and Technology, Zhuhai, 519082, China’ but should have been ‘Sino-French Institute of Nuclear Engineering and Technology, Sun Yat-sen University, Zhuhai, 519082, China’. The original article has been corrected.

