# Supplementary material for: The unusual quadruple bonding of nitrogen in ThN
Source: Nat Commun. 2023 Nov 24;14:7677. doi: 10.1038/s41467-023-43208-z (PMC10667236; doi:10.1038/s41467-023-43208-z)
Supplement: Supplementary file 3 — Supplementary information [file 41467_2023_43208_MOESM3_ESM.pdf]

Supplementary Information

The Unusual Quadruple Bonding of Nitrogen in ThN

Zejie Fei[1,7], Jia-Qi Wang[2,3,7], Rulin Tang[4], Yuzhu Lu[4], Changcai Han[1], Yongtian Wang[1], Jing Hong[1,5], Changwu Dong[1], Han-Shi Hu[2], Xiao-Gen Xiong[*6], Chuangang Ning[*4], Hongtao Liu[*1], Jun Li[2]

[1] Key Laboratory of Interfacial Physics and Technology, Shanghai Institute of Applied Physics, Chinese Academy of Sciences, Shanghai 201800. China.

[2] Department of Chemistry, Tsinghua University, Beijing 10084, China

[3] College of Science, Beijing Forestry University, Beijing 10083, China

[4] Department of Physics, State Key Laboratory of Low Dimensional Quantum Physics, Collaborative Innovation Center of Quantum Matter, Tsinghua University, Beijing 10084, China

[5] University of Chinese Academy of Sciences, Beijing 100049. China

[6] Sun Yat-sen University, Sino-French Institute of Nuclear Engineering and Technology, Zhuhai 519082, China

[7] These authors contributed equally: Zejie Fei, Jia-Qi Wang

Correspondence and requests for materials should be addressed to Xiao-Gen Xiong (E-mail: xiongxg@mail.sysu.edu.cn), Hongtao Liu (E-mail: liuhongtao@sinap.ac.cn) or Chuangang Ning (E-mail: ningcg@tsinghua.edu.cn)

Supplementary Computational Methods:

We used both the density functional theory (DFT) and high-level wave function theory (WFT) to study the various molecules investigated in this article. The geometries have been fully optimized at both DFT level and WFT level. All the DFT and WFT calculations were performed using Gaussian 16[1] and Molpro2020.2[2], respectively. In order to accurate generate the theoretical bond length, we optimized the geometries of the studied molecules at the CCSD(T) (coupled-cluster singles-and-doubles plus perturbative triples) level[3]. In the Gaussian and Molpro calculations, the scalar-relativistic effects of thorium were considered through the pseudopotential approach, in which the energy-consistent pseudopotential ECP60MDF (Th) [4] of Stuttgart/Cologne group and the corresponding valence triple-ζ basis sets[5] were used. The all-electron augmented triple-ζ basis sets aug-cc-pVTZ were used for C and N[6]. We hereafter will call AVTZ (for C and N) and VTZ-PP (for Th) for the basis sets employed in Gaussian and Molpro calculations. Compared with DFT calculation, the accuracy of coupled cluster theory is very sensitive to the size of basis sets, therefore we further performed the CCSD(T) calculations using augmented quadruple-ζ basis sets aug-cc-pVQZ for C and N[6], quadruple-ζ basis sets cc-pVQZ-PP for Th[4, 5]. Here we will abbreviate the quadruple-ζ basis sets used in this study as AVQZ for C and N, and VQZ-PP for Th, respectively. The NBO calculations were performed using NBO 7.0 package[7] to generate the Weinhold's natural localized molecular orbitals, where the wavefunction of each molecule is generated at the level of TPSS exchange-correlation functional[8] accompanied with triple-ζ basis sets.

We also performed the DFT calculations with PBE exchange-correlation functional[9] implemented in Amsterdam Density Functional (ADF 2016.01) [10] to optimize the geometries and confirm the minimum through vibrational frequency calculations. In ADF calculations, the scalar-relativistic (SR) effects were handled through zero-order-regular approximation (ZORA) [11], and the Kohn-Sham molecular orbital (MO) energies based on PBE SR-ZORA optimized geometry using the statistical average of orbital potentials (SAOP) [12] were directly used in depicting the correlation diagrams of neutral diatomic molecule. We also used the ADF to do the EDA-NOCV[13] calculations for the neutral ground states of ThC and ThN. We used Slater basis sets with the quality of triple-ζ plus two polarization functions (TZ2P) in ADF calculations, and for geometry optimization and vibrational frequency calculations, we applied the frozen core approximation for all elements, i.e., [1s^2-5d^{10}] for Th and [1s^2] for C and N.

In order to incorporate the spin-orbit (SO) effects, the low-lying excited states of the neutral ThC and ThN were calculated at the SO-CASPT2 level[14], in which the SO effects are treated perturbatively. The similar approach has been widely used in predicting the electronic structures of molecules containing heavy elements in our previous work. Specifically, in our calculations, in order to generate the accurate detachment energies of the studied anion, we calculated the excited states of neutral molecules at the CCSD(T) optimized anion geometries. The state-specific SO-averaged CASPT2 energy for each target state was calculated based on the state-averaged complete active space self-consistent field (SA-CASSCF) reference function. The active space in our calculations

including19 MOs with significant contributions of 2*s*- and 2*p*-AOs of C/N, and the 5*f*-, 6*d*-, 7*s*- and 7*p*-AOs of thorium. In the CASPT2 calculations, an empirical correction, named ionization potential electron affinity (IPEA) shift[15] with a value of 0.20 a.u., was applied to the zeroth-order Hamiltonian. The purpose of IPEA shift is reducing the systematic error which can lead to a relative overestimation of the correlation energy for open-shell molecules. The level shift technique[16] with 0.30 a.u. was also used to avoid intruder states and improve the convergence.

Supplementary Table 1 Optimized bond length at the level of PBE, PBE0, B3LYP, TPSS, TPSSh and CCSD(T) for ThC^-, ThC, ThN^- and ThN. AVTZ for C, N, and VTZ-PP basis sets were used for all DFT and CCSD(T) calculations. For CCSD(T) calculations we also used quadruple-level basis sets.

	$R_{(Th–C)}$		$R_{(Th–N)}$	
	ThC^-	ThC	ThN^-	ThN
PBE	1.971	1.934	1.850	1.819
PBE0	1.959	1.923	1.832	1.802
B3LYP	1.977	1.939	1.848	1.816
TPSS	1.976	1.938	1.850	1.820
TPSSh	1.970	1.933	1.842	1.813
CCSD(T)[a]	1.986	1.951	1.857	1.822
CCSD(T)[b]	1.985	1.948	1.854	1.820

a. VTZ-PP for Th, AVTZ for C and N;

b. VQZ-PP for Th, AVQZ for C and N.

Supplementary Table 2 Low-lying SO-CASPT2 state energies for ThC at the CCSD(T) optimized anion geometry. Triple-ζ basis sets (AVTZ for C, VTZ-PP for Th) were used in SO-CASPT2 calculation, and quadruple-ζ basis sets were used in CCSD(T) calculation.

Energy order	$\Delta E/cm^{-1}$	ΔE/eV	SO State	Degeneracy (Spatial)	Composition of SF states
1	0	0.0000	$^3\Sigma_0^+$	1	97%$^3\Sigma^+$
2	6	0.0007	$^3\Sigma_1^+$	2	98%$^3\Sigma^+$
3	1149	0.1425	$^1\Sigma_0^+$	1	97%$^1\Sigma^+$
4	4831	0.5990	$^3\Pi_2$	2	100%$^3\Pi$
5	5068	0.6284	X_0	1	67%$^3\Pi$ + 31%$^1\Sigma^+$
6	5210	0.6460	$^3\Pi_1$	2	96% $^3\Pi$
7	5721	0.7094	$^3\Pi_0$	1	97%$^3\Pi$
8	6321	0.7836	X_0	1	61% $^1\Sigma^+$+39%$^3\Pi$

* X_0 state is the mixing of $^1\Sigma^+$ and $^3\Pi$, which makes it difficult to characterize.

Supplementary Table 3 Low-lying SO-CASPT2 state energies for ThN at the CCSD(T) optimized anion geometry. Triple-ζ basis sets (AVTZ for N, VTZ-PP for Th) were used in SO-CASPT2 calculation, and quadruple-ζ basis sets were used in CCSD(T) calculation.

Energy order	$\Delta E/cm^{-1}$	$\Delta E/eV$	SO State	Composition of SF states
1	0	0.0000	$^2\Sigma^+_{1/2}$	99.7% $^2\Sigma^+$
2	8726	1.0882	$^2\Delta_{3/2}$	99.6% $^2\Delta$
3	11132	1.3865	$^2\Delta_{5/2}$	100% $^2\Delta$
4	11283	1.4052	$^2\Sigma^+_{1/2}$	100% $^2\Sigma^+$
5	15676	1.9499	$^2\Pi_{1/2}$	99.6% $^2\Pi$
6	17905	2.2262	$^2\Pi_{3/2}$	99.6% $^2\Pi$

Supplementary Table 4 Leading symmetrized fragment orbital (SFO) percentage contribution of Kohn-Sham orbitals for ThC and ThN. The theoretical results are based on statistical SAOP calculations

	ThC
19σ	78.11% Th-7s + 9.95% Th-6d_{σ} + 7.04% Th-7p_{σ} + 2.49% C-2p_{σ}
18σ	52.80% C-2p_{σ} + 21.29% Th-6d_{σ} + 14.86% Th-6p_{σ} + 5.20% Th-5f_{σ}
10π	52.46% C-2p_{π} + 27.90% Th-6d_{π} + 7.25% Th-5f_{π}
17σ	74.14% C-2s + 12.91% Th-6d_{σ} + 6.88% Th-6p_{σ} + 4.88% Th-7s + 2.34% Th-5f_{σ}
	ThN
19σ	81.80% Th-7s + 7.54% Th-7p_{σ} + 7.15% Th-6d_{σ} + 2.07% N-2p_{σ}
18σ	55.81% N-2p_{σ} + 16.68% Th-6d_{σ} + 11.84% Th-7p_{σ} + 6.78% Th-5f_{σ} + 2.96% Th-6p_{σ}
10π	64.00% N-2p_{π} + 27.90% Th-6d_{π} + 7.25% Th-5f_{π}
17σ	65.88% N-2s + 18.61% Th-6p_{σ} + 10.30% Th-6d_{σ} + 2.93% Th-7s + 2.25% Th-5f_{σ}

* Fractional occupations were used for singly occupied orbitals.

Supplementary Table 5 Atomic hybrid contributions of four NLMOs on Th-X (X = C and N). All results are based on TPSS calculations. All electron basis sets AVTZ were used for C and N, while VTZ-PP was employed for Th.

ThC		
NLMO	Spin-α	Spin-β
σ1	50% Th($s^{0.07}d^{0.67}f^{0.26}$) + 50% C($s^{0.22}p^{0.78}$)	-
π	39% Th($d^{0.86}f^{0.14}$) + 61% C($p^{1.00}$)	37% Th($d^{0.81}f^{0.19}$) + 63% C($p^{1.00}$)
π′	39% Th($d^{0.86}f^{0.14}$) + 61% C($p^{1.00}$)	37% Th($d^{0.81}f^{0.19}$) + 63% C($p^{1.00}$)
σ2	1% Th($s^{0.09}p^{0.23}d^{0.19}f^{0.48}$) + 99% C($s^{0.82}p^{0.22}$)	17% Th($s^{0.29}d^{0.61}f^{0.10}$) + 83% C($s^{1.00}$)
ThN		
NLMO	Spin-α	Spin-β
σ1	40% Th($s^{0.04}p^{0.01}d^{0.64}f^{0.30}$) + 60% N($s^{0.19}p^{0.81}$)	40% Th($d^{0.65}f^{0.35}$) + 60% N($s^{0.18}p^{0.82}$)
π	28% Th($p^{0.78}f^{0.22}$) + 72% N($s^{0.02}p^{0.98}$)	25% Th($d^{0.75}f^{0.25}$) + 75% N($p^{1.00}$)
π′	28% Th($p^{0.78}f^{0.22}$) + 72% N($s^{0.02}p^{0.98}$)	25% Th($d^{0.75}f^{0.25}$) + 75% N($p^{1.00}$)
σ2	1% Th($s^{0.08}p^{0.43}d^{0.20}f^{0.29}$) + 99% N($s^{0.84}p^{0.16}$)	3% Th($s^{0.82}p^{0.05}d^{0.05}f^{0.08}$) + 97% N($s^{0.85}p^{0.15}$)

Supplementary Table 6 Theoretical atomic charges on Th and the calculated bond orders of Th–X (X = C, N)

	Net Charge			
	Hirshfeld	VDD	MDC-q	Bader
ThC	0.444	0.557	1.307	0.944
ThN	0.522	0.578	1.398	1.281
	Bond Order			
	Mayer	G-J	N-M(3)	
ThC	2.96	3.08	4.00	
ThN	3.10	3.05	4.20	

ThC ($^{3}\Sigma^{+}$)		ThN ($^{2}\Sigma^{+}$)	
22σ<br>LUMO+7	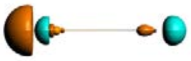	22σ<br>LUMO+7	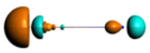
21σ<br>LUMO+6	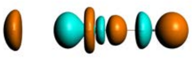	21σ<br>LUMO+6	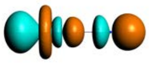
6δ<br>LUMO+5	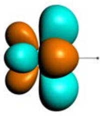	12π<br>LUMO+5	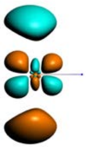
12π<br>LUMO+4	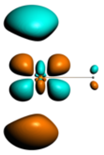	6δ<br>LUMO+4	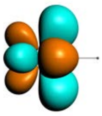
2ϕ<br>LUMO+3	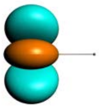	2ϕ<br>LUMO+3	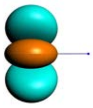
20σ<br>LUMO+2	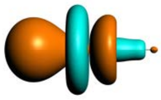	20σ<br>LUMO+2	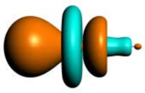
11π<br>LUMO+1	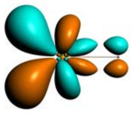	11π<br>LUMO+1	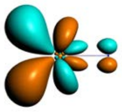
5δ<br>LUMO	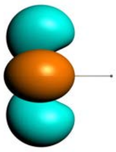	5δ<br>LUMO	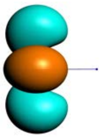
19σ<br>SOMO	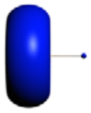	19σ<br>SOMO	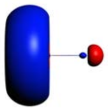
18σ<br>SOMO	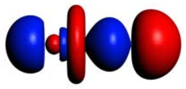	18σ<br>HOMO	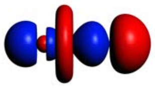
10π<br>HOMO	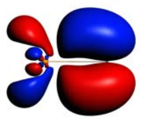	10π<br>HOMO-1	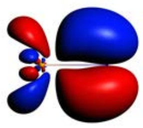
17σ<br>HOMO-1	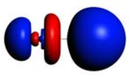	17σ<br>HOMO-2	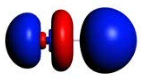

Supplementary Figure 1 Contour surfaces of Kohn-Sham MOs of ThC and ThN (isosurface = 0.5 a.u.). Source data are provided as a Source data file.

Supplementary References

1 Gaussian 16 Rev. B.01 (Wallingford, CT, 2016).

2 MOLPRO 2020.2, a package of *ab initio* programs, Werner, H.-J., Knowles, P. J., and others, see https://www.molpro.net.

3 Watts, J. D., Gauss, J. & Bartlett, R. J. Coupled‐cluster methods with noniterative triple excitations for restricted open‐shell Hartree–Fock and other general single determinant reference functions. Energies and analytical gradients. *J. Chem. Phys.* **98**, 8718-8733 (1993).

4 Weigand, A., Cao, X., Hangele, T. & Dolg, M. Relativistic Small-Core Pseudopotentials for Actinium, Thorium, and Protactinium. *J. Phys. Chem. A* **118**, 2519-2530 (2014).

5 Peterson, K. A. Correlation consistent basis sets for actinides. I. The Th and U atoms. *J. Chem. Phys.* **142**, 074105 (2015).

6 Dunning, T. H. J. Gaussian basis sets for use in correlated molecular calculations. I. The atoms boron through neon and hydrogen. *J. Chem. Phys.* **90**, 1007-1023 (1989).

7 NBO 7.0. Glendening, E. D., Badenhoop, J. K., Reed, A. E., Carpenter, J. E., Bohmann, J. A., Morales, C. M., Karafiloglou, P., Landis, C. R., and Weinhold, F., Theoretical Chemistry Institute, University of Wisconsin, Madison, WI (2018).

8 Tao, J., Perdew, J. P., Staroverov, V. N. & Scuseria, G. E. Climbing the Density Functional Ladder: Nonempirical Meta--Generalized Gradient Approximation Designed for Molecules and Solids. *Phys. Rev. Lett.* **91**, 146401 (2003).

9 Perdew, J. P., Burke, K. & Ernzerhof, M. Generalized Gradient Approximation Made Simple. *Phys. Rev. Lett.* **77**, 3865-3868 (1996).

10 ADF 2016.01, SCM, Theoretical Chemistry, Vrije Universiteit, Amsterdam, The Netherlands (http://www.scm.com).

11 Lenthe, E. v., Ehlers, A. & Baerends, E.-J. Geometry optimizations in the zero order regular approximation for relativistic effects. *J. Chem. Phys.* **110**, 8943-8953 (1999).

12 Chong, D. P., Gritsenko, O. V. & Baerends, E. J. Interpretation of the Kohn–Sham orbital energies as approximate vertical ionization potentials. *J. Chem. Phys.* **116**, 1760-1772 (2002).

13 Mitoraj, M. P., Michalak, A. & Ziegler, T. A Combined Charge and Energy Decomposition Scheme for Bond Analysis. *J. Chem. Theory Comput.* **5**, 962-975 (2009).

14 Roos, B. O. & Malmqvist, P.-Å. Relativistic quantum chemistry: the multiconfigurational approach. *Phys. Chem. Chem. Phys.* **6**, 2919-2927 (2004).

15 Ghigo, G., Roos, B. O. & Malmqvist, P.-Å. A modified definition of the zeroth-order Hamiltonian in multiconfigurational perturbation theory (CASPT2). *Chem. Phys. Lett.* **396**, 142-149 (2004).

16 Roos, B. O. & Andersson, K. Multiconfigurational perturbation theory with level shift — the Cr_2 potential revisited. *Chem. Phys. Lett.* **245**, 215-223 (1995).
