# Supplementary material for: The unusual quadruple bonding of nitrogen in ThN
Source: Nat Commun. 2023 Nov 24;14:7677. doi: 10.1038/s41467-023-43208-z (PMC10667236; doi:10.1038/s41467-023-43208-z)
Supplement: Supplementary file 4 — Source Data [file 41467_2023_43208_MOESM4_ESM.zip › SourceData_Fig.1c_2c.pptx]

## Slide 1
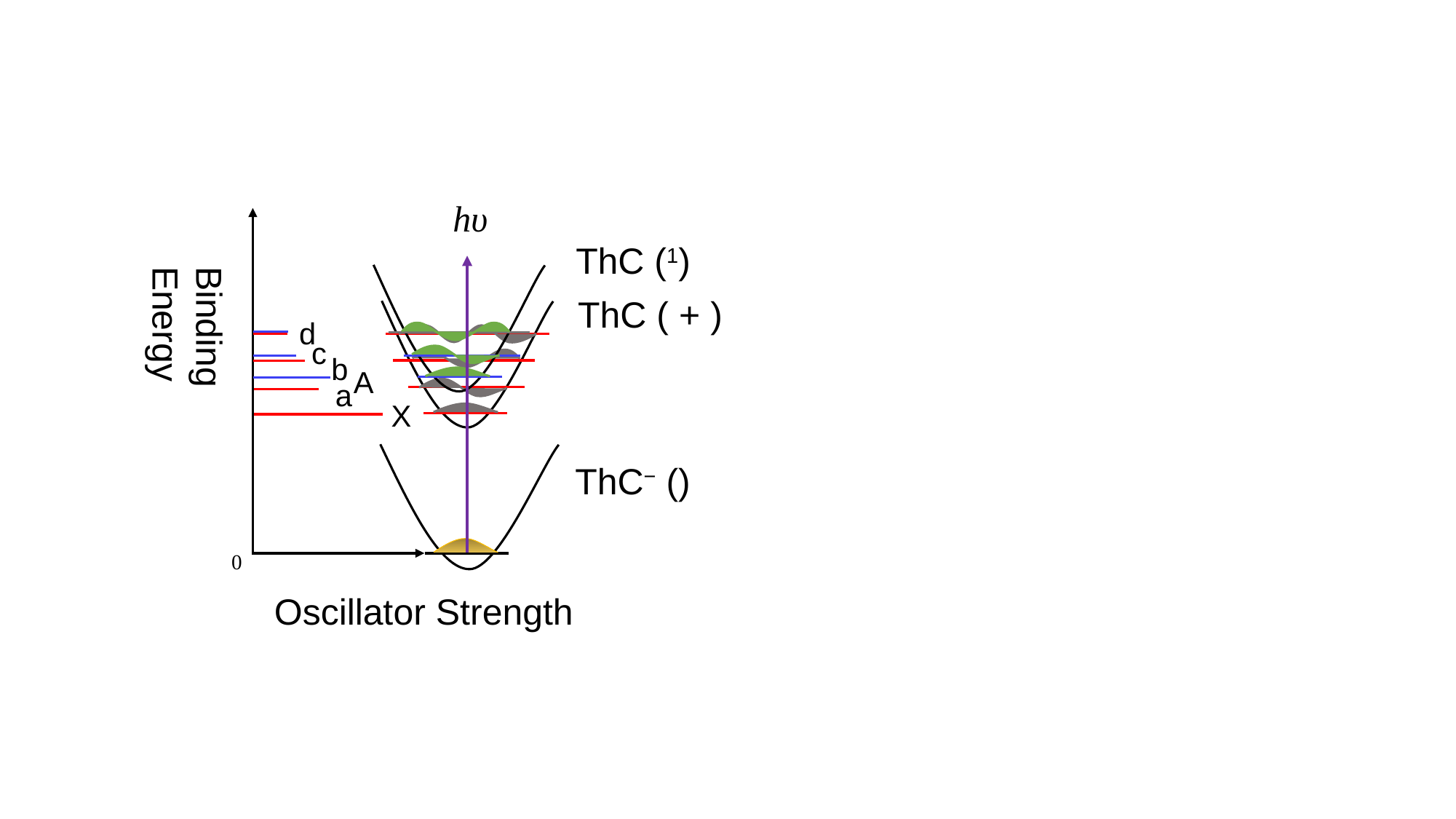

hυ
d
c
b
A
a
X
0
Binding Energy
Oscillator Strength

## Slide 2
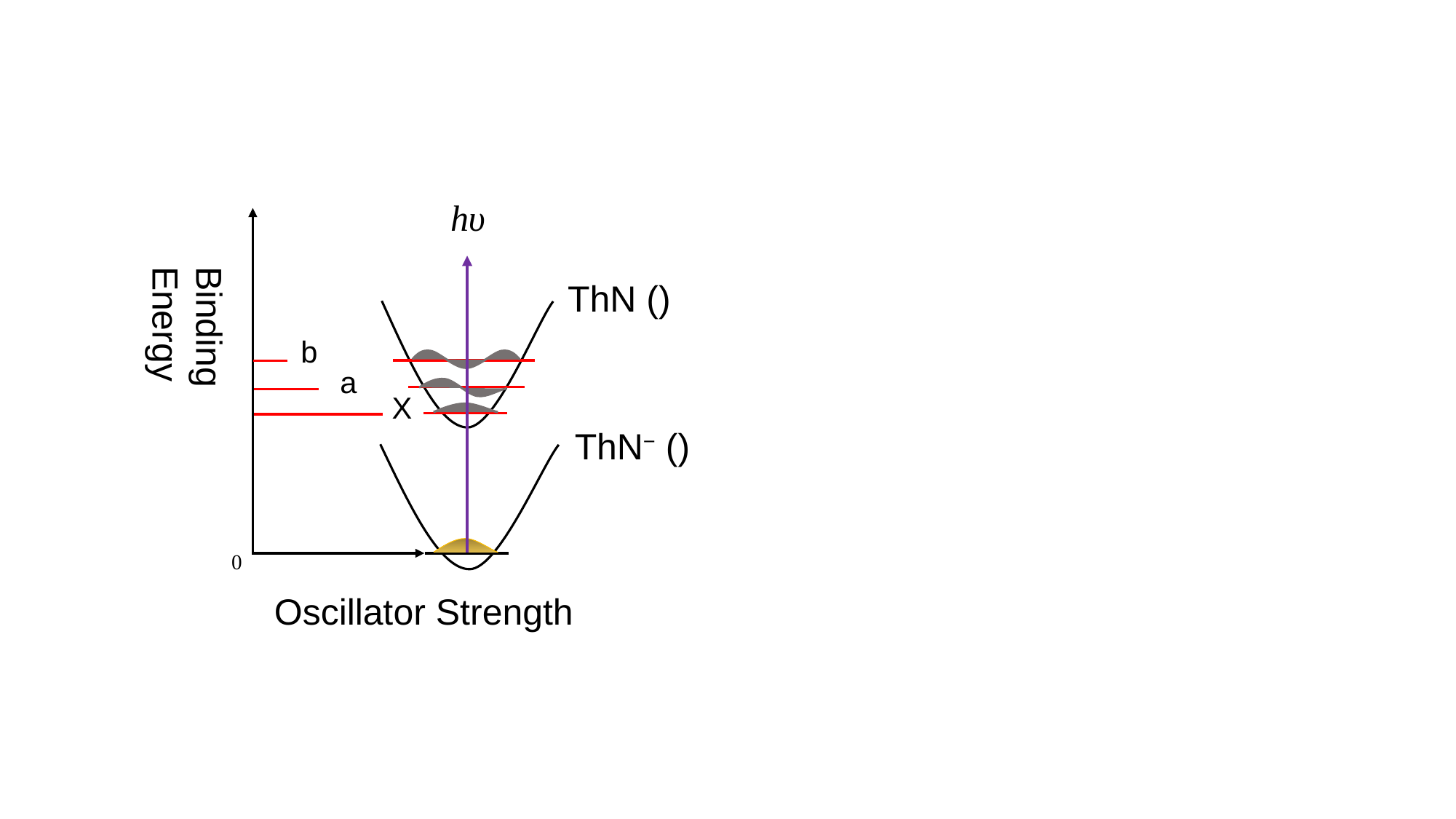

hυ
0
Binding Energy
b
a
X
Oscillator Strength
